# Remote physiologic monitoring for hypertension in primary care: a prospective pragmatic pilot study in electronic health records using propensity score matching

**DOI:** 10.1093/jamiaopen/ooac111

**Published:** 2023-01-31

**Authors:** Lucia C Petito, Lauren Anthony, Yaw Amofa Peprah, Ji Young Lee, Jim Li, Hironori Sato, Stephen D Persell

**Affiliations:** Division of Biostatistics, Department of Preventive Medicine, Feinberg School of Medicine, Northwestern University, Chicago, Illinois, USA; Northwestern Medical Group Quality and Patient Safety, Northwestern Memorial Healthcare, Chicago, Illinois, USA; Division of General Internal Medicine, Department of Medicine, Feinberg School of Medicine, Northwestern University, Chicago, Illinois, USA; Division of General Internal Medicine, Department of Medicine, Feinberg School of Medicine, Northwestern University, Chicago, Illinois, USA; Clinical Development Department, Technology Development HQ, Omron Healthcare, Co., Ltd, Kyoto, Japan; Product Innovation Department, Technology Development HQ, Omron Healthcare, Co., Ltd, Kyoto, Japan; Division of General Internal Medicine, Department of Medicine, Feinberg School of Medicine, Northwestern University, Chicago, Illinois, USA; Center for Primary Care Innovation, Institute for Public Health and Medicine, Feinberg School of Medicine, Northwestern University, Chicago, Illinois, USA

**Keywords:** remote physiologic monitoring, blood pressure, hypertension management

## Abstract

**Objectives:**

Since 2019, the Centers for Medicare and Medicaid Services covers remote physiologic monitoring (RPM) for blood pressure (BP) per hypertension diagnosis and treatment guidelines. Here, we integrated Omron VitalSight RPM into the health system’s electronic health record to transmit BP and pulse without manual entry, assessed feasibility, and used pragmatic prospective matched cohort studies to assess initial effects in (1) uncontrolled (last two office BP ≥140/90 mmHg) and (2) general (diagnosed hypertension or last office BP ≥140/90 mmHg) hypertension patient populations.

**Materials and Methods:**

Seventeen clinicians at two internal medicine practices were oriented. Eligible patients were aged 65–85 years had Medicare insurance with ≥1 office visit in the previous year. We prospectively identified matched controls (age, sex, BP, and number of office visits in previous year) from other primary care practices within the health system and estimated the association between RPM availability (clinic-level) and patient BP outcomes after 6 months. ClinicalTrials.gov: NCT04604925.

**Results:**

*Feasibility.* Uptake was low at pilot clinics: 10 physicians prescribed RPM to 118 patients during the 6-month pilot. This included 7% (14/207) of the prespecified uncontrolled hypertension cohort and 3.3% (78/2356) of the general hypertension cohort. Surveyed clinicians (*n *= 4) reported changing their patients’ medical treatment in response to RPM BPs, although they recommended having a dedicated RN or LPN to review BP readings. *Effectiveness.* At 6 months, BP control was greater at pilot practices than among matched controls (uncontrolled: 31.4% vs 22.8%; *P* = .007; general: 64.0% vs 59.7%; *P* < .001). Systolic BP at last office visit did not differ (mean [SD] 146.0 [15.7] vs 147.1 [15.6]; *P* = .48) in the uncontrolled population, and was lower in the general population (131.8 [15.7] vs 132.8 [15.9]; *P* = .04).The frequency of antihypertensive medication changes was similar in both groups (uncontrolled *P* = .986; general *P* = .218).

**Discussion and Conclusions:**

Uptake notwithstanding, RPM may have improved BP control. A potential mechanism is increased physician awareness of and attention to uncontrolled hypertension. Barriers to RPM use among physicians require further study.

## BACKGROUND AND SIGNIFICANCE

Hypertension is a major contributor to death and disability.^1^ Despite extensive healthcare contact, most hypertensive individuals do not have their blood pressure (BP) controlled.^1^^,^^2^ Regular home BP measurements may be a better indicator of cardiovascular risk than infrequent office BP measurements and can identify both masked hypertension and white coat hypertension.^3–5^ The evidence-based 2017 joint American College of Cardiology (ACC)/American Heart Association (AHA) hypertension guidelines recommend using out-of-office BP measurements to confirm diagnosis and titrate BP-lowering medications in conjunction with other clinical interventions.^6–8^ Home BP monitoring coupled with clinical team members who can intensify hypertension care has led to lower achieved BPs and better hypertension control.^4^^,^^5^^,^^9–12^ This body of work demonstrates improvements that can be made in hypertension control using home monitoring-based interventions, but further investigation on how to extend these approaches into routine clinical care is needed.

In November 2019, the Centers for Medicare and Medicaid Services added coverage for remote physiologic monitoring (RPM) services that can be used to support hypertension management using home monitoring.^13^ Cost has been identified previously as a barrier to RPM usage, by patients and health systems alike, so this reimbursement could alleviate financial concerns and mutually benefit both parties.

## OBJECTIVES

Here, we conducted a pragmatic pilot study where we integrated an RPM system for BP directly into a health system’s electronic health record (EHR) and made RPM available at two primary care practices. Study aims were to (1) assess the feasibility of implementing RPM into the EHR and routine care via an implementation study and a clinician user experience survey and (2) add to preliminary research assessing the effectiveness of RPM in two patient groups: the uncontrolled hypertensive population and the general hypertensive population using a prospective matched cohort design.

## MATERIALS AND METHODS

This study describes the implementation of an RPM system for BP and the initial uptake, usage, and results of making this capability available to physicians caring for Medicare-enrolled patients at two primary care internal medicine practices from November 18, 2020 through May 17, 2021. We solicited user experience feedback from physicians via electronic survey. Furthermore, we compared BPs between cohorts of hypertensive patients from these practices and matched control patients from other primary care practices within the Northwestern Medical Group (NMG) in northeastern Illinois. NMG uses one EHR, Epic (Epic Systems Corp., Verona, WI), from which all study data, including data used for matching criteria and outcome data, were abstracted. See Supplementary Table S1 for identification of study variables. This study was approved by the Northwestern University Institutional Review Board and the system-wide primary care working group. The analysis plan was prespecified and is available at ClinicalTrials.gov: NCT04604925. Clinical data used in this study were obtained in the course of routine medical care and used with a waiver of informed consent.

### RPM feasibility study

#### Implementation

We integrated Omron VitalSight RPM into NMG’s EHR to automatically transmit BP and pulse data to patients records at two *a priori* selected primary care practices.

To setup RPM at Northwestern Medicine for the transmission of BP, pulse, and weight data, Northwestern Medicine and Redox (Redox, Inc. Madison, WI), a third-party platform, integrated a VPN to send/receive HL7v2 messages. Prior to integration, we obtained review and approval from several Northwestern Medicine committees and departments (Primary Care Health System Collaborative, Application Rationalization, Security Review, Technical Assessment, Data Stewardship, and Clinical Decision Support Committee), and established Business Associate Agreements (BAA) with the vendors. The integration allowed for orders to be transmitted from the Northwestern Medicine EHR to Redox, and then to Omron, and for physiologic measurement data to transmit directly from the patient’s BP cuff to a cellular transmitter, to Omron, to Redox, and then into the patients EHR record. Once ordered, patients were mailed a kit, which contained automated BP monitor, cellular data hub, and optional scale. In Basket messages to prescribing clinicians were delivered on a regular schedule based on number of readings and days elapsed. Clinicians also received a real-time message for values within the adjustable alerting range, defaulted to values outside of 90–180 mmHg systolic or 40–110 mmHg diastolic.

In this study, we made available RPM for BP and weight, as well as treatment management services allowable under Medicare, to patients at the selected practices when ordered by the patient’s clinician. These practices were selected for regional representation (one from each of the central and north regions); the first two clinics approached agreed to participate. All primary care clinicians at intervention practices received communication by email and at practice meetings explaining RPM procedures, ordering, use, and financial implications. Clinical decision support in the form of an Epic “Best Practice Advisory” notified clinicians when patients met study criteria for RPM for 6 months (November 18, 2020 through May 17, 2021) (Supplementary Figure S1). It remained at the discretion of the clinicians whether to offer RPM. Clinicians were encouraged to tell their patients to take home readings based on the recommendations of the American Heart Association.^14^ How often home measurements were transmitted to the ordering clinician’s inbox could be selected at the time of ordering. We did not dictate to clinicians how often or how long they should tell their patients to monitor. Readings with systolic blood pressure (SBP) below 90 or above 180 or diastolic below 40 or above 110 were transmitted to the clinician inbox immediately and clinicians could adjust these thresholds.

#### Participants

**Figure 1.**
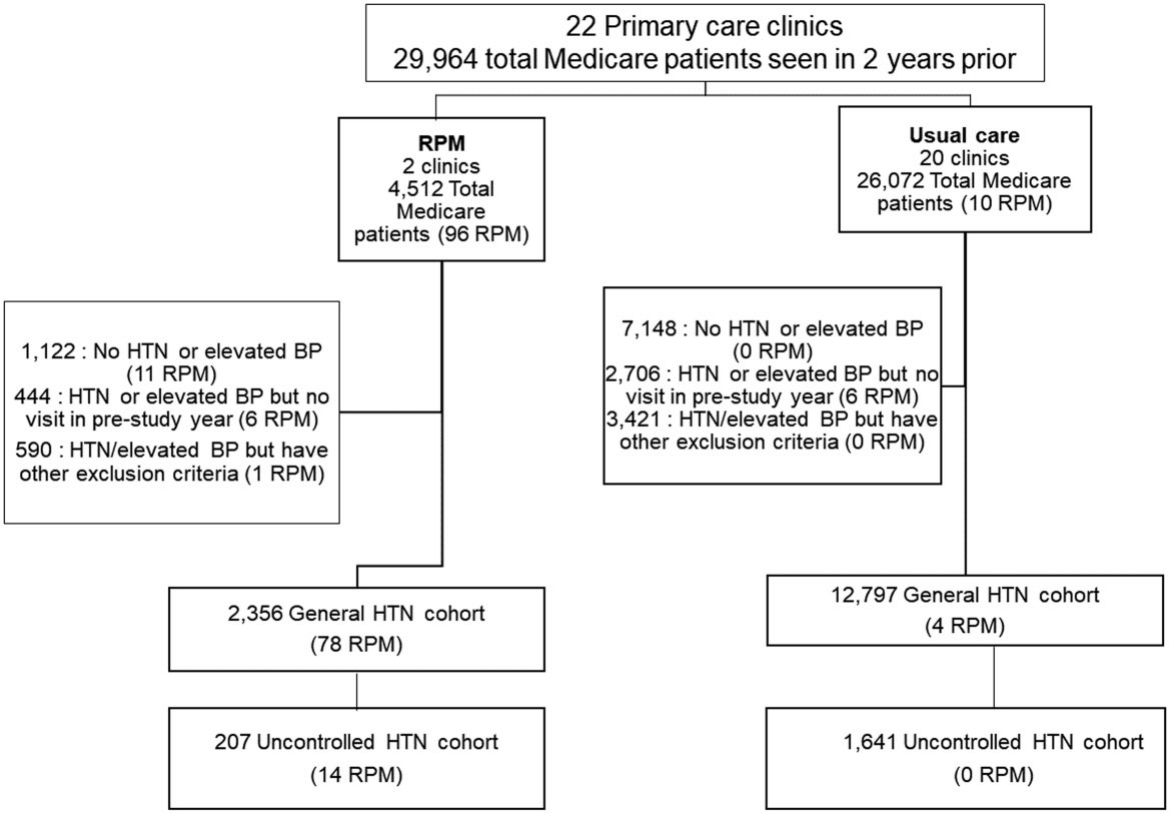
Diagram demonstrating eligibility of Medicare patients at the 2 primary care practices with RPM available and 20 usual care practices, November 2018–November 2020. Hypertension cohorts were defined prior to outcome ascertainment, which occurred November 2020–May 2021. Primary care clinicians could prescribe RPM to any Medicare patient including those not included in the predefined cohorts and could prescribe it for diagnoses other than hypertension (eg, heart failure). Ten patients from usual care clinics were prescribed RPM from an endocrinology practice that was not part of this study. Abbreviation: RPM: remote physiologic monitoring.

Two cohorts were of interest: patients with uncontrolled hypertension, and the general hypertensive population. The uncontrolled hypertension cohort had elevated BP at the two most recent in-office primary care encounters (≥140/90 mmHg) *and* a diagnosis of hypertension in the year prior to November 17, 2020. The general hypertensive population included all individuals in the uncontrolled hypertension cohort, plus individuals with a diagnosis of hypertension with controlled BP (<140/90 mmHg) at one of the two most recent in-office primary care encounters, and individuals without a hypertension diagnosis but with elevated BP (≥140/90 mmHg) at the most recent primary care encounter. All individuals were required to be age 65–85 years old, have Medicare or Medicare Advantage insurance, have at least one office or telehealth visit at an eligible primary care site in the year prior to November 17, 2020, and no evidence of persistent or permanent atrial fibrillation, stage IV or more severe kidney disease, or diagnosed dementia. Figure 1 visualizes eligible patient selection.

*Primary outcomes: feasibility metrics*. Data from patients of clinicians at pilot practices were abstracted from the EHR. To understand utilization, we measured the number of physicians who prescribed RPM devices, and the number of patients prescribed BP and weight RPM devices. Among those individuals, we measured whether they used the devices, time to uptake (days between prescription and first reading), and the intensity of use (mean BP readings per week). We also measured the number of office visits, patient portal messages, and telephone encounters. We counted the frequency of CPT codes associated with RPM (99453, 99454, 99457, 99458) to quantify RPM billing.

*Secondary outcomes: blood pressure measurements*. To understand the impact of RPM on BP, we assessed the Controlling High BP performance measure as defined by the 2020 National Quality Forum Measure 0018 (NQF0018): most recent BP reading <140/90 mmHg.^15^^,^^16^ This widely used measure uses the lowest SBP and diastolic blood pressure (DBP) obtained on the day with the most recent BP measurement, and includes in-office BPs and BPs obtained via RPM directly transmitted to the healthcare provider. To ascertain the sensitivity of this public performance measure to remote measurements, we created a version that only used office BPs. Other secondary outcomes included most recent in-office SBP and DBP, and most recent SBP and DBP with the inclusion of remote readings. For these, if an individual had multiple in-office or RPM BP measurements available on a particular day, we used their average as the “most recent” BP. We also counted the net number of hypertensive medication intensifications, the number of antihypertensive drugs added or increased minus the number discontinued or decreased, via an algorithm that accounted for drug class and dosing.

#### Statistical analysis

We described patient characteristics and healthcare usage in RPM enrollees. To explore overall BP trends in frequent RPM users (≥12 RPM BPs transmitted in the study period), we visualized patient trajectories of RPM BPs, stratified by whether the patient had controlled BP (<140/90 mmHg) at their most recent in-office visit. We used stratified linear regression as a function of time since RPM prescription to overlay a smoothed line on each plot and visualize the daily change in RPM-reported BPs.

### Elucidating the role of RPM in detangling white coat and masked hypertension

To understand how RPM measurements can affect the prevalence of white coat and masked hypertension, we calculated the average RPM BPs obtained within 2 weeks of the index date. We varied the time windows for a sensitivity analysis, comparing BP measurements from the most recent primary care office visit within 1 year prior to the study end to the most recent transmitted RPM BP reading collected during the study period. As the definition of uncontrolled BP was recently updated, users were classified as at-home controlled if this BP reading was (1) <130/80 mmHg or (2) <135/85 mmHg, according to current AHA guidelines and prior cutpoints.^8^^,^^17^^,^^18^ We calculated the prevalence of masked hypertension (individuals with uncontrolled RPM BP and controlled in-office BP) and white coat hypertension (individuals with controlled RPM BP and uncontrolled in-office BP).

### Clinician user experience survey

We surveyed pilot physicians’ about experiences with RPM for BP. This included questions about their likelihood to use RPM, barriers to and satisfaction with implementation of RPM, and physician characteristics. The survey was disseminated via email, and encrypted data capture occurred through Northwestern University’s instance of REDCap. All clinicians at both intervention clinics received an initial email containing the survey request, with two reminders sent 1 week apart.

#### Statistical analysis

Responses to survey questions were summarized; proportions were calculated for questions with two or three answers, and median accompanied by the range was used for continuous variables.

### Preliminary assessment of RPM effectiveness via matched cohort study

#### Participants and measurements

To approximate an intention-to-treat effect from a randomized clinical trial and avoid potential selection bias induced by differential preference for beginning RPM, we chose to include all patients of physicians at clinics where RPM was available in our effectiveness study. We then selected two cohorts, uncontrolled and general hypertensive, of “usual care” matched controls using propensity scores from patients who received primary care at 20 other NMG practices. Cohorts were then prospectively followed for 6 months (November 18, 2020 through May 17, 2021). As the RPM device was piloted in primary care practices, we only used data collected at primary care appointments to facilitate exchangeability between intervention patients matched controls. All BP metrics were abstracted from the EHR and calculated as described above.

#### Statistical analysis

To compare BP outcomes between patients at intervention practices and those receiving primary care elsewhere, we created two propensity score-matched cohorts to balance baseline characteristics. *A priori* selected matching factors included: sex, age, SBP, DBP, and number of BP measurements (0, 1, 2, 3+) in the 0–5 months and 6–12 months prior to study start date. We abstracted these EHR data the week before the study start date. We implemented a greedy matching algorithm that incorporated a logistic regression-generated propensity score; the model predicted being seen at an RPM-eligible clinic with the matching factors as predictors.^19^ The number of matches was selected based on visual inspection of the propensity score distribution (Supplementary Figures S2 and S3); we chose 4:1 for the uncontrolled hypertension cohort and 2:1 for the broader hypertension cohort.

We described the baseline demographic characteristics, healthcare usage, and clinical presentation in the matched cohorts. At the end of the 6-month follow-up, we calculated adjusted averages and proportions of outcomes for RPM-eligible patients and matched controls. Generalized linear models were used to estimate differences in means of continuous variables (identity link) and differences in log-odds for categorical variables (logit link); a type-I error rate of 5% was prespecified. All models were adjusted for the matching characteristics (indicator variables for categorical, linear terms for continuous).

Some BP data (24–28% in months 0–5; 31–40% in months 6–12) were missing for matching factors; these data were singly imputed (Supplementary Table S2). All analyses were done in R version 4.0.3 (R Core Team); matching was done via the ‘MatchIt’ package and imputation via the ‘missForest’ package.

## RESULTS

### RPM feasibility study

Of 17 primary care physicians from two pilot practices, 10 physicians ordered RPM for 118 patients (Table 1). RPM-enrollees were 75 years of age on average, 66% female, 86% non-Hispanic White, and 98% had chronic hypertension. The diagnosis linked to the RPM order was essential hypertension for all but three patients. Uptake of RPM was variable. Twenty-five (21%) patients never transmitted remote BP measurements. Of the patients who ever transmitted a BP measurement, most people used the device frequently—91% transmitted at least 12 remote BP measurements. The median user transmitted their first BP measurement 8 days after the order, and transmitted 9.5 measurements per week. All but one patient had at least one in-person/telehealth visit during the study period. Sixty-two percent of the RPM-prescribed patients had billing codes generated. There were 73 and 138 instances of RPM CPT billing codes 99453 and 99454, respectively. These were generated automatically when RPM usage criteria were met. None of the clinicians billed the care management codes (99457, 99458).

**Table 1.** Characteristics of patients enrolled in remote patient monitoring at pilot practices: November 2020–May 2021

	RPM-enrolled patients
Patients, *n*	118
Practices, *n*	2
Primary care physicians, *n*	10
RPM-enrolled patients per physician, median (IQR)	5.5 (2, 9)
Characteristics of enrolled patients	
Age, mean (SD)	75.2 (6.5)
Female, *n* (%)	78 (66.1)
Site, *n* (%)	
Site A	27 (22.9)
Site B	91 (77.1)
Race, *n* (%)	
White	103 (87.3)
Black	11 (9.3)
Other/declined/unknown	4 (3.4)
Hispanic or Latino ethnicity, *n* (%)	1 (0.9)
Primary language: English, *n* (%)	117 (99.2)
Baseline clinical characteristics	
Chronic conditions, *n* (%)	
Atrial fibrillation	13 (11.0)
Chronic kidney disease	9 (7.6)
Coronary heart disease	15 (12.7)
Diabetes mellitus	23 (19.5)
Heart failure	6 (5.1)
Hypertension	116 (98.3)
Most recent in-office SBP^a^ in year prior to study start, mean (SD)	134.4 (18.0)
Most recent in-office DBP^a^ in year prior to study start, mean (SD)	75.1 (8.7)
Controlling high blood pressure: Most recent BP^b^ <140/90 mmHg in year prior to study start, *n* (%)	64 (54.2)
Diagnosis code associated with RPM order, *n* (%)	
Essential hypertension	115 (97.5)
Elevated blood pressure reading	1 (0.8)
Hypertensive heart and kidney disease/CKD (chronic kidney disease)	1 (0.8)
Hypertensive renal disease	1 (0.8)
Clinical outcomes after 6 months	
Controlling high blood pressure: Most recent BP^c^ <140/90 mmHg (office or RPM), *n* (%)	82 (69.5)
Controlling high blood pressure: Most recent BP^c^ <140/90 mmHg (office only), *n* (%)	61 (51.7)
Most recent SBP^d^ (office only), mean (SD)	141.4 (20.4)
Most recent SBP^d^ (office or RPM), mean (SD)	133.2 (17.9)
Most recent DBP^d^ (office only), mean (SD)	77.0 (9.4)
Most recent DBP^d^ (office or RPM), mean (SD)	75.6 (9.5)
Prescription intensification change, *n* (%)	
No change	73 (61.9)
Any decrease	13 (11.0)
Any increase	32 (27.1)
Healthcare utilization	
At least 1 in-person office visit within 1 year of study end date, *n* (%)	114 (96.6)
In-person/telehealth encounters during the study period, *n* (%)	
0	1 (0.9)
1	67 (56.8)
2	27 (22.9)
3+	23 (19.5)
Patient portal encounters during the study period, *n* (%)	
0	33 (28.0)
1	24 (20.3)
2	10 (8.5)
3+	51 (43.2)
Telephone encounters during the study period, *n* (%)	
0	35 (29.7)
1	29 (24.6)
2	13 (11.0)
3+	41 (34.8)
CPT^16^ Billing code frequency, *n*	
99453: initial set up and patient education of monitoring equipment	73
99454: device(s) supply with daily recording(s) each 30 days	138
99457: remote physiologic monitoring treatment management services, 20 min or more	0
RPM usage	
Total number of remote BP transmitted, *n*	8974
Patients with any remote BP transmitted, *n* (%)	93 (78.8)
Patients with at least 12 remote BP transmitted, *n* (%)	85 (72.0)
Time to initial use (days), median (IQR)	8 (6, 16)
Number of remote BP transmitted per week (intensity), median (IQR)	9.5 (5.2, 15)
Patients with RPM scale prescribed, *n* (%)	67 (56.8)
Patients with any remote weight transmitted, *n* (%)	57 (48.3)
Total number of weights remotely transmitted	2826

Abbreviations: RPM: remote physiologic monitoring; IQR: inner quartile range; SD: standard deviation; SBP: systolic blood pressure; DBP: diastolic blood pressure; BP: blood pressure.

a When multiple blood pressure measurements were available in a time window, their average was used.

b When multiple blood pressure measurements were available on the same day, the lowest SBP and lowest DBP were used according to the measure specifications. “Most recent” was defined as within 1 year of study start (November 19, 2019–November 18, 2020).

c When multiple blood pressure measurements were available on the same day, the lowest SBP and lowest DBP were used according to the measure specifications. “Most recent” was defined as within 1 year of study end (May 19, 2020–May 18, 2021).

d When multiple blood pressure measurements were available on the same day, their average was used. “Most recent” was defined as within 1 year of study end (May 19, 2020–May 18, 2021).

**Figure 2.**
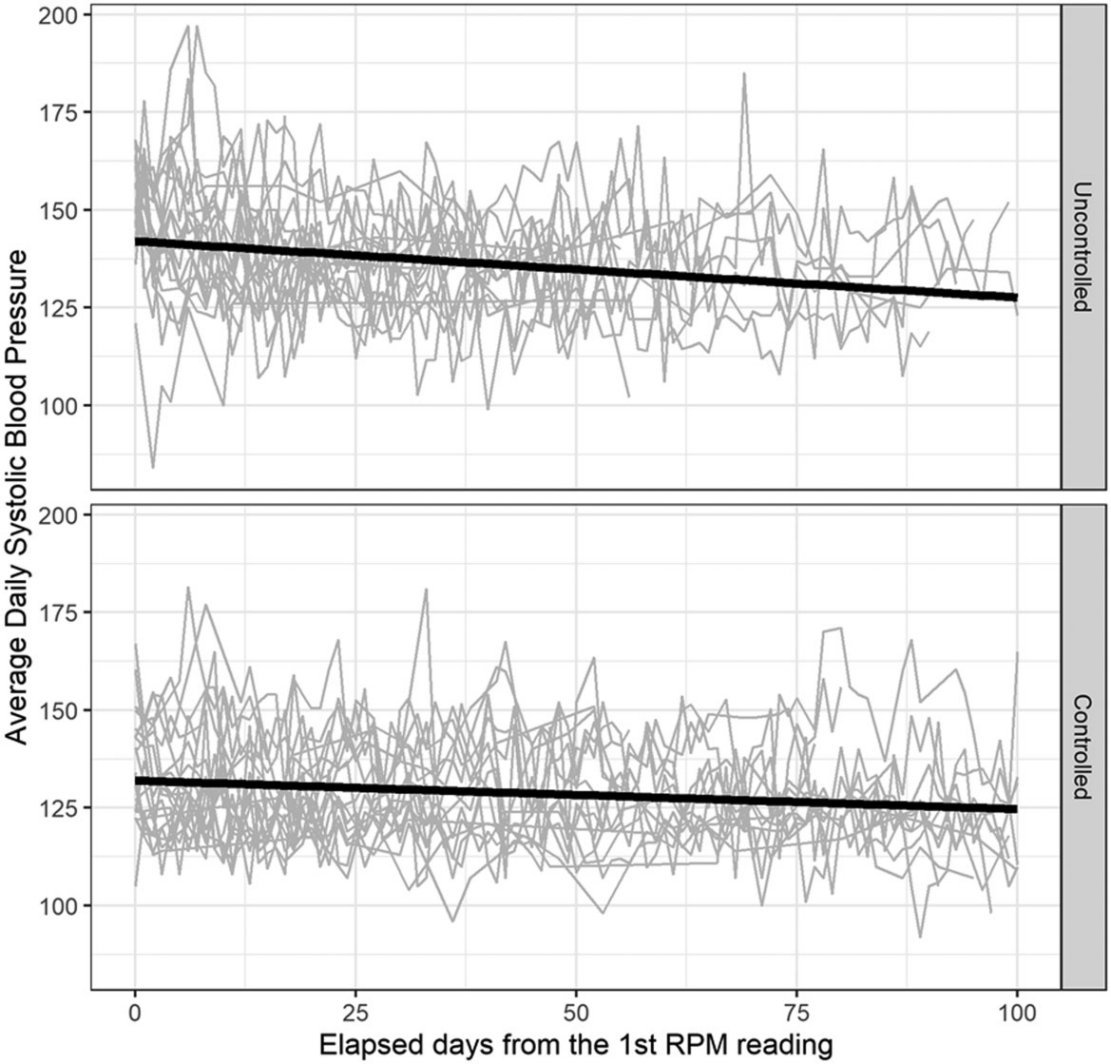
First 100 days of systolic blood pressure readings among 40 randomly selected patients who transmitted at least 12 remote readings during the study period, stratified by most recent in-office BP before initial RPM reading, November 2020–May 2021. “Controlled” was defined as having a BP reading <140/90 mmHg at the most recent in-person office visit prior to the initial RPM reading. Grey lines represent patient-specific trajectories of the first 100 days of systolic BP RPM readings among a subset of patients who transmitted at least 12 BPs via RPM during the study period. Bold lines represent results from linear regression of all participants who transmitted at least 12 remote readings; the slopes of these lines represent the daily change in average systolic blood pressures. For uncontrolled patients, this slope was −0.14 mmHg/day; for controlled patients, this slope was −0.07 mmHg/day. Abbreviations: BP: blood pressure; RPM: remote physiologic monitoring.

**Figure 3.**
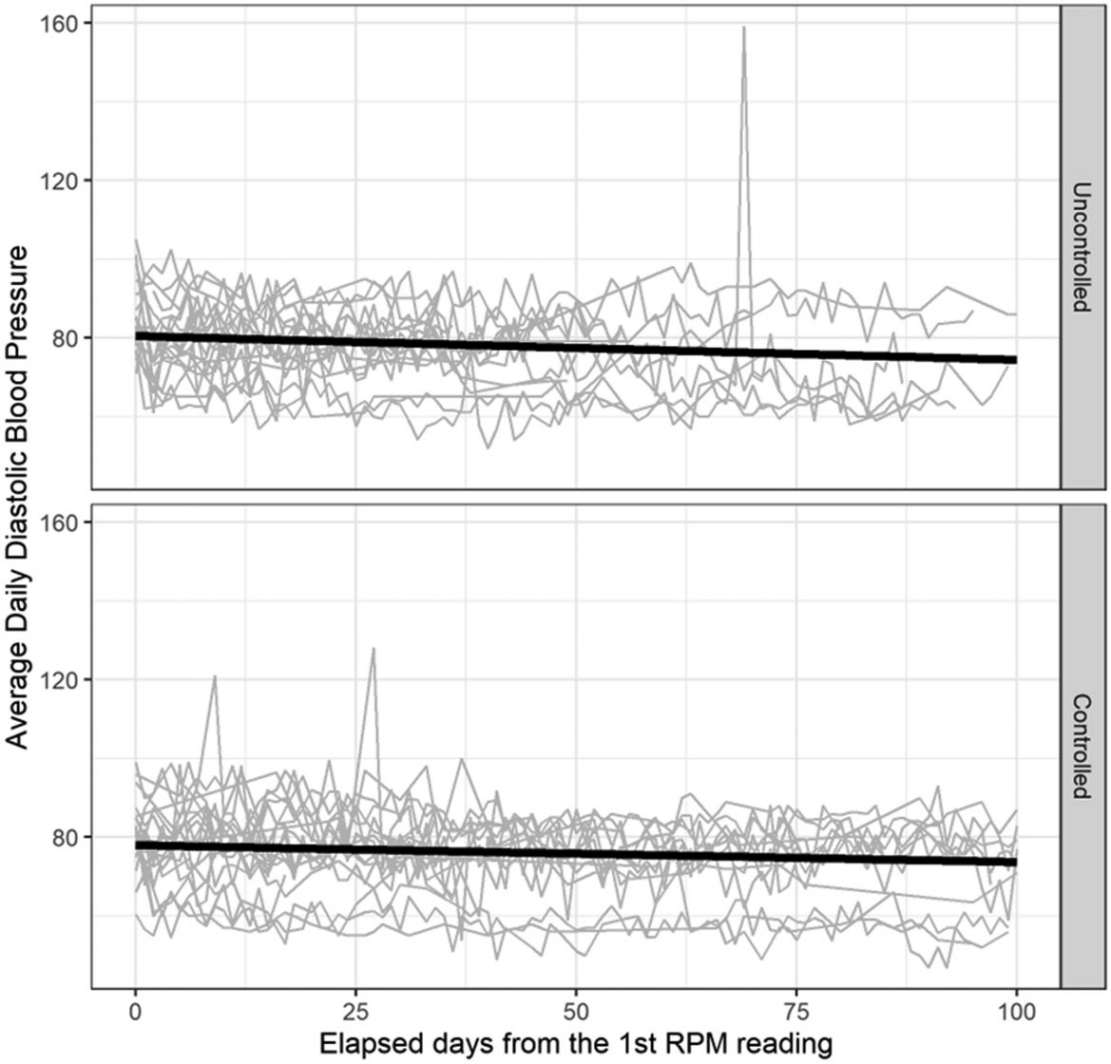
First 100 days of diastolic blood pressure readings among 40 randomly selected patients who transmitted at least 12 remote readings during the study period, stratified by most recent in-office BP before initial RPM reading, November 2020–May 2021. “Controlled” was defined as having a BP reading <140/90 mmHg at the most recent in-person office visit prior to the initial RPM reading. Grey lines represent patient-specific trajectories of the first 100 days of diastolic BP RPM readings among a subset of patients who transmitted at least 12 BPs via RPM during the study period. Bold lines represent results from linear regression of all participants who transmitted at least 12 remote readings; the slopes of these lines represent the daily change in average diastolic blood pressures. For uncontrolled patients, this slope was −0.06 mmHg/day; for controlled patients, this slope was −0.04 mmHg/day. Abbreviations: BP: blood pressure; RPM: remote physiologic monitoring.

SBPs and DBPs from RPM readings trended downwards over the study period in both uncontrolled and controlled RPM users (Figures 2 and 3). At baseline, 54% of RPM users satisfied the Controlling High BP metric using in-office BP measurements, with an average most recent BP reading of 135/75 mmHg (Table 1). After 6 months, 70% of RPM users satisfied this metric using both in-office or RPM measurements, with an average most recent BP reading of 133/76 mmHg. Interestingly, measures using only in-office BP measurements closely resemble those from study baseline: 52% under control, with an average most recent BP reading of 141/77. While 62% of RPM users had no change in their antihypertensive medication, 27% experienced a net increase, and 11% had a net decrease.

### Elucidating the role of RPM in detangling white coat and masked hypertension

We compared in-office versus remote BP measurements among the subgroup of 63 RPM users who transmitted at least 12 readings during the study period and had remote values within 2 weeks before or after an office visit (Table 2). Among these patients, 84% (53/63) had concordance between their office and remote BPs at the <130/80 mmHg cut-point. 6% of patients had apparent white-coat hypertension or white-coat effect, 10% of patients had apparent masked hypertension or masked uncontrolled hypertension. Using the criteria for control of <135/85 mmHg home, <140/90 mmHg office, concordance was 67% (42/63), 13% of patients had white-coat, and 21% of patients had masked hypertension.

**Table 2.** Comparison of in-office^a^ versus remote^b^ BP measurements among remote monitoring patients who transmitted at least 12 remote readings during the study period and had remote values within 2 weeks before or after an office visit using (A) AHA 2017 criteria and (B) older criteria: November 2020–May 2021^1^^,^^6^^,^^12^^,^^13^

(A) AHA criteria				
		RPM measurement^b^		
		≥130/80	<130/80	Total
In-office measurement^a^	≥130/80	47 (74.6%)	4 (6.4%)	51
	<130/80	6 (9.5%)	6 (9.5%)	12
	Total	53	10	63

(B) Older criteria				
		RPM measurement^b^		
		≥135/85	<135/85	Total
In-office measurement^a^	≥140/90	21 (33.3%)	8 (12.7%)	29
	<140/90	13 (20.6%)	21 (33.3%)	34
	Total	34	29	63

Abbreviations: AHA: American Heart Association; RPM: remote physiologic monitoring.

a When multiple blood pressure measurements were available on the same day, their minimum was used. Only in-office visits during the study period were included.

b Value used was mean of all remote BP measurements transmitted 14 days before or after the office visit.

### Clinician user experience survey

Of 17 eligible clinicians, 4 (24%) internal medicine physicians with, on average, more than 2 decades’ experience responded to the survey request (Supplementary Table S4). Three (75%) physicians reported they ordered RPM and received BP readings in Epic. One physician never ordered RPM.

#### Clinician user experience

On a scale of 1–10 (lowest to highest), three physicians would recommend RPM for other primary care practices (median 7, range 1–10), albeit with minor changes/improvements such as “dedicated RN or LPN to review BP readings” and “making it easier to place an order for RPM device”. Some physicians felt that RPM results returned too often (*n* = 3/4), although they thought it was helpful to receive extreme values immediately (*n* = 2/4) and they could easily tell if a patient’s hypertension was controlled (*n* = 3/4).

#### Patient care experience

All three physicians who ordered RPM reported that they had communicated directly with a patient in the patient portal because of RPM and had asked a patient to schedule an earlier follow-up appointment in response to RPM BP values. Furthermore, two (of 3) physicians changed at least one patient’s medical treatment and telephoned a patient (or had a nurse do so) in response to RPM BP values.

#### Future prescribing of RPM

Three physician prescribers stated they would be likely to order RPM for additional patients with uncertain BP control, and two of three indicated they would be very likely to order RPM for patients with uncontrolled BP. Importantly, three (out of 4) physicians reported that they would be unlikely to order RPM for patients whose BP is controlled in the office but unknown at home.

### Preliminary assessment of RPM effectiveness via matched cohort study

#### Uncontrolled hypertensive cohort

We identified 207 patients with uncontrolled hypertension seen by doctors at the two pilot practices and matched them to 828 patients at 20 other primary care practices. Matching produced similar groups (Table 3). There were more non-White patients at intervention practices than controls (45% vs 30%).

**Table 3.** Baseline patient characteristics for uncontrolled hypertensive and general hypertensive matched cohorts: November 2019–November 2020

	Uncontrolled hypertensive			General hypertensive		
	Pilot practices	Matched controls^a^	Standardized mean difference	Pilot practices	Matched controls^a^	Standardized mean difference
No. of patients	207	828	–	2356	4712	–
No. of practices	2	20	–	2	20	–
Demographics						
Age, mean (SD)	73.6 (5.4)	73.6 (5.2)	0.012	73.1 (5.1)	73.2 (5.1)	0.02
Female, *n* (%)	146 (70.5)	582 (70.3)	0.029	1554 (66.0)	3119 (66.2)	0.009
Race, *n* (%)			–			–
White	113 (54.6)	580 (70.1)		1551 (65.8)	3410 (72.4)	
Black	68 (32.9)	131 (15.8)		564 (23.9)	662 (14.1)	
Asian	9 (4.4)	28 (3.4)		80 (3.4)	208 (4.4)	
Other	10 (4.8)	52 (6.3)		101 (4.3)	236 (5.0)	
Declined/Unknown	7 (3.4)	37 (4.5)		60 (2.6)	196 (4.2)	
Ethnicity, *n* (%)			–			–
Hispanic or Latino	8 (3.9)	48 (5.8)		87 (3.7)	246 (5.2)	
Non-Hispanic or Latino	192 (92.8)	721 (87.1)		2164 (91.9)	4161 (88.3)	
Declined/Unknown	7 (3.4)	59 (7.1)		105 (4.5)	305 (6.5)	
Primary language, *n* (%)			–			–
English	203 (98.1)	742 (89.6)		2317 (98.3)	4495 (95.4)	
Spanish	2 (1.0)	23 (2.8)		14 (0.6)	83 (1.8)	
Other	2 (1.0)	63 (7.6)		25 (1.1)	134 (2.8)	
Clinical characteristics						
Chronic conditions, *n* (%)						
Atrial fibrillation (paroxysmal)	16 (7.7)	55 (6.6)	–	163 (6.9)	420 (8.9)	–
Chronic kidney disease	15 (7.2)	69 (8.3)		202 (8.6)	308 (6.5)	
Coronary heart disease	21 (10.1)	116 (14.0)	–	382 (16.2)	823 (17.5)	–
Diabetes mellitus	57 (27.5)	251 (30.3)	–	631 (26.8)	1249 (26.5)	–
Heart failure	13 (6.3)	37 (4.5)	–	146 (6.2)	229 (4.9)	–
Hypertension	204 (98.6)	818 (98.8)	–	2201 (93.4)	4304 (91.3)	–
Average SBP^b^ 0–5 months prior to study start, mean (SD)	151.7 (10.1)	151.6 (10.8)	0.005	131.3 (14.7)	131.4 (15.0)	0.006
Average DBP^b^ 0–5 months prior to study start, mean (SD)	79.7 (8.1)	79.7 (9.1)	0.005	74.9 (8.3)	74.9 (8.8)	0.018
No. of BP measurements 0–5 months prior to study start, *n* (%)			0.043			0.003
0	54 (26.1)	196 (23.7)		651 (27.6)	1259 (26.7)	
1	79 (38.2)	362 (43.7)		1129 (47.9)	2337 (49.6)	
2	53 (25.6)	183 (22.1)		445 (18.9)	793 (16.8)	
3+	21 (10.1)	87 (10.5)		131 (5.6)	323 (6.9)	
Average SBP^b^ 6–12 months prior to study start, mean (SD)	148.5 (12.2)	148.4 (11.5)	0.003	131.3 (14.1)	131.5 (13.9)	0.011
Average DBP^b^ 6–12 months prior to study start, mean (SD)	79.7 (8.4)	79.4 (8.6)	0.028	74.8 (8.0)	75.1 (8.3)	0.001
No. of BP measurements 6–12 months prior to study start, *n* (%)			0.005			0.012
0	64 (30.9)	277 (33.5)		910 (38.6)	1903 (40.4)	
1	90 (43.5)	336 (40.6)		1030 (43.7)	1975 (41.9)	
2	41 (19.8)	153 (18.5)		328 (13.9)	584 (12.4)	
3	12 (5.8)	62 (7.5)		88 (3.7)	250 (5.3)	
Controlling high blood pressure: Most recent BP^c^ <140/90, *n* (%)	0 (0)	0 (0)	–	1674 (71.1)	3175 (67.4)	–
Healthcare utilization						
No. office and telehealth encounters per patient within 1 year of study start, *n* (%)			0.08			0.007
1	43 (20.8)	175 (21.1)		710 (30.1)	1555 (33.0)	
2	53 (25.6)	243 (29.4)		704 (29.9)	1244 (26.4)	
3–4	86 (41.5)	270 (32.6)		697 (29.6)	1292 (27.4)	
5+	25 (12.1)	140 (16.9)		245 (10.4)	621 (13.2)	

Abbreviations: No.: number; SD: standard deviation; SBP: systolic blood pressure; DBP: diastolic blood pressure; BP: blood pressure.

a Matched controls were selected from the uncontrolled and general hypertensive patient populations at nonpilot practices using 4:1 and 2:1 propensity score matching, respectively. The algorithm included: age, sex, average SBP, and DBP in 0–5 and 6–12 months prior to study start, and # of in-person or telehealth visits in 0–5 and 6–12 months prior to study start.

b When multiple blood pressure measurements were available in a time window, their average was used.

c When multiple blood pressure measurements were available on the same day, the lowest SBP and lowest DBP were used according to the measure specifications. “Most recent” was defined as within 1 year of study start (November 19, 2019–November 18, 2020).

Table 4 shows BP measurements and healthcare use in each study arm at the end of the 6-month follow-up period. Of the 207 patients at the intervention practices, 14 (6.8%) of them had RPM ordered during the study period. More patients at the pilot practices satisfied the Controlling High BP metric that incorporated both in-office and RPM BP measurements (31% vs 23%, *P* = .007). Restricting to only in-office measurements reduces the number of patients at the pilot practices who satisfied the Controlling High BP metric (29% vs 23%, *P* = .047). We did not observe a clinically meaningful difference in the most recent SBP and DBP measurements (using in-office and RPM values) (145 vs 147, *P* = .18; 77 vs 78, *P* = .08). Similarly, we did not see a difference in prescription intensification between study arms (no change/increase in 75%/18% vs 77%/17% of patients, *P* = .99).

**Table 4.** Remote patient monitoring use and blood pressure outcomes during 6-month study follow-up in uncontrolled and general hypertensive matched cohorts: November 2020–May 2021

	Uncontrolled hypertensive cohort			General hypertensive cohort		
	Pilot practices	Matched controls^a^	*P* value^b^	Pilot practices	Matched controls^a^	*P* value^b^
No. of patients	207	828	–	2356	4712	–
No. of practices	2	20	–	2	20	–
RPM ordered^c^, *n* (%)	14 (6.8)	0 (0)	–	78 (3.3)	4 (0.1)	–
Clinical outcomes						
Controlling high blood pressure: Most recent BP^d^ <140/90 mmHg (office or RPM), *n* (%)	65 (31.4)	189 (22.8)	.0066	1508 (64.0)	2813 (59.7)	.0001
Controlling high blood pressure: Most recent BP^d^ <140/90 mmHg (office only), *n* (%)	60 (29.0)	189 (22.8)	.0469	1495 (63.5)	2812 (59.7)	.0008
Controlling high blood pressure: Most recent BP^d^ <140/90 mmHg (office or RPM) among patients with an in-person office visit within 12 months of study end, *n* (%)	65 (36.1)	189 (26.2)	.0071	1507 (73.5)	2812 (68.1)	<.0001
Controlling high blood pressure: Most recent BP^d^ <140/90 mmHg (office only) among patients with an in-person office visit within 12 months of study end, *n* (%)	60 (33.3)	189 (26.2)	.0522	1495 (72.9)	2813 (68.2)	<.0001
Most recent SBP^e^^,^^f^ (office or RPM), mean (SD)	145.3 (16.6)	147.1 (15.6)	.1818	131.6 (15.5)	132.8 (15.9)	.0077
Most recent SBP^f^ (office only), mean (SD)	146.0 (15.7)	147.1 (15.6)	.4839	131.8 (15.7)	132.8 (15.9)	.0396
Most recent DBP^f^ (office or RPM), mean (SD)	76.8 (10.0)	78.1 (10.0)	.0771	74.5 (8.8)	75.0 (9.0)	.0210
Most recent DBP^f^ (office only), mean (SD)	76.8 (9.6)	78.1 (10.0)	.0705	74.5 (8.7)	75.0 (9.0)	.0286
Prescription intensification change, *n* (%)			.9857			.2184
No change	156 (75.4)	634 (76.6)		1933 (82.1)	3876 (82.3)	
Any decrease	14 (6.8)	53 (6.4)		123 (5.2)	281 (6.0)	
Any increase	37 (17.9)	141 (17.0)		300 (12.7)	555 (11.8)	
Healthcare system usage						
At least one in-person office visit within 12 months of study end, *n* (%)	180	721	–	2051	4127	–
In-person encounters per patient during the study period^g^, *n* (%)			–			–
0	55 (26.6)	291 (35.1)		726 (30.8)	1788 (38.0)	
1	76 (36.7)	260 (31.4)		958 (40.7)	1667 (35.4)	
2	48 (23.2)	149 (18.0)		421 (17.9)	779 (16.5)	
3+	28 (13.5)	128 (15.5)		251 (10.7)	478 (10.1)	
Patient portal encounters per patient during the study period^g^, *n* (%)			–			–
0	110 (53.1)	392 (47.3)		1135 (48.2)	1996 (42.4)	
1	31 (15.0)	119 (14.4)		409 (17.4)	764 (16.2)	
2	21 (10.1)	80 (9.7)		263 (11.2)	543 (11.5)	
3+	45 (21.7)	237 (28.6)		549 (23.3)	1409 (29.9)	
Telephone encounters per patient during the study period^g^, *n* (%)			–			–
0	114 (55.1)	338 (40.8)		1397 (59.3)	2011 (42.7)	
1	43 (20.8)	151 (18.2)		466 (19.8)	995 (21.1)	
2	20 (9.7)	97 (11.7)		190 (8.1)	534 (11.3)	
3+	30 (14.5)	242 (29.2)		303 (12.9)	1172 (24.9)	

Abbreviations: BP: blood pressure; DBP: diastolic blood pressure; RPM: remote physiologic monitoring; SBP: systolic blood pressure.

a Matched controls were selected from the uncontrolled and general hypertensive patient populations at nonpilot practices using 4:1 and 2:1 propensity score matching, respectively. The algorithm included: age, sex, average SBP and DBP in 0–5 and 6–12 months prior to study start, and # of in-person office visits in 0–5 and 6–12 months prior to study start.

b Reported *P* values for binary and continuous outcomes are from logistic and linear regression models, respectively. All models were adjusted for the matching variables.

c Patients were only eligible for the matched cohort if they had data available in the 2 years prior to study start. The discrepancy between the number of RPM ordered in this table versus the number reported in Table 1 is likely due to new patients joining the healthcare system, or older patients with a longer gap in care. Four of the matched controls received RPM from an endocrinologist who was investigating RPM outside this pilot.

d When multiple blood pressure measurements were available on the same day, the lowest SBP and lowest DBP were used according to the measure specifications. “Most recent” was defined as within 1 year of study end (May 19, 2020–May 18, 2021).

e Primary study endpoint. “Most recent” was defined as within 1 year of study end (May 19, 2020–May 18, 2021).

f When multiple blood pressure measurements were available on the same day, the average was used.

g Study period ran November 19, 2020 through May 18, 2022.

#### General hypertensive cohort

We identified 2356 patients in the broader hypertensive population from pilot practices and matched them to 4712 patients at the other practices (Table 3). There were more Black patients at intervention practices than matched controls (24% vs 14%). The baseline BP and the prevalence of chronic conditions were similar across study arms.

Seventy-eight (3.3%) of the 2356 patients at intervention practices and 4 (0.1%) of the 4712 matched controls had RPM ordered during the study period (Table 4). More patients at the pilot practices satisfied Controlling High BP (64% vs 60%, *P* < .001) and there were small differences in the most recent SBP and DBP measurements (132 vs 133, *P* < .01; 74.5 vs 75.0, *P* = .02). The interpretation did not change after excluding RPM BP measurements. We did not observe a difference in the prevalence of antihypertensive medication intensification between study arms (82% in each arm had no change in antihypertensive medication intensity, *P* = .22); 13% of the pilot group and 12% of the matched control group experienced a net increase in antihypertensive therapy.

## DISCUSSION

This prospective matched cohort study is, to our knowledge, the first study to describe implementation of RPM of BP in primary care practices utilizing the recently covered Medicare benefit. Although RPM was available, uptake was quite modest: over a 6-month period, 10 physicians ordered RPM for 118 patients—including 6.8% of the predefined poorly controlled hypertension cohort and 3.3% of the general hypertension cohort. That said, RPM availability affected physician behavior, as 38% (45/118) of patients prescribed RPM had a net change in antihypertensive medications during the study, contrasted with 24% (51/207) of the uncontrolled and 17% (423/2356) of the general hypertensive patient populations at pilot clinics. Possibly as a result, the proportion of patients prescribed RPM meeting the Controlling High BP measure rose from 54.2% (64/118) at the start to 69.5% (82/118) by the end of the study.

Among the 63 high-use RPM patients who transmitted BP data within 2 weeks of an office visit, we saw concordance between in-office and RPM BP measurements among 84% (53/63) of patients using AHA 2017 criteria, and only 67% (42/63) using the older criteria that uses higher cut points and different BP levels to indicate hypertension in and out of the office. As we expected, we detected white coat effect as well as masked hypertension. The proportion of patients with BP <130/80 mmHg, the current ACC/AHA guideline, was low both in and out of the office, demonstrating the need for ongoing efforts to improve hypertension management.

Patients in both the uncontrolled and general hypertension cohorts at practices offering RPM were more likely to satisfy the Controlling High BP quality measure at 6-months compared to matched controls, and we quantified the extent to which these differences were due to the inclusion of RPM BP values. Among the general hypertensive cohort, we observed small differences in SBP and DBP by the study end (approximately 1/0.5 mmHg) whether or not we restricted the observations to office-only BPs. We did not observe meaningful differences in medication intensification in either of the matched cohorts, suggesting that differences in the Controlling High BP metric may be due to factors other than differences in medication intensification.

These findings have implications for public reporting. We observed improvements in NQF0018 which includes out-of-office BP values in the measure. This finding may incentivize stakeholders who are motivated to improve performance on this metric to promote the use of RPM.

RPM was available to 17 clinicians with patient panels including over 2000 patients with hypertension or elevated BP. There was substantial variability in RPM prescribing—seven physicians never-prescribed and four prescribed over 80% of orders. Despite efforts to improve survey participation, there was a low response rate (4/17, 24%, Supplementary Table S4). Although our ability to suggest improvements to increase clinician uptake is limited, reasons identified for not using RPM by all four respondents included concern for cost to the patient and that the patient was already self-monitoring. Patients with traditional Medicare who did not have supplemental coverage that included RPM services were responsible for 20% of the charges. Areas for potential improvement include clarifying out-of-pocket costs to patients up front and identifying nonphysician staff to review incoming results. Despite orienting physicians to the billing components, none in this group charged for the clinician management services associated with RPM. Improving reimbursement for the clinical work done performing RPM may be important to the sustainability of such programs.

### Strengths and limitations

The major study strength was leveraging the EHR for a prospective design. We established matched cohorts and followed them for 6 months. Using the EHR to collect all study data helped to ensure systematic collection, minimize loss to follow-up, and reduce bias from data self-reporting.

The main limitation was that randomization was not feasible. Intervention practices were *a priori* selected, so these patients and physicians could be different from those elsewhere in NMG. The matched design should alleviate much of this bias, but residual unmeasured confounding is possible, particularly if the behaviors of the clinicians at the sites participating in this pilot differ from those of the other sites. Second, we observed fairly low uptake of RPM, and only small percentages of the intervention cohorts received RPM. Greater RPM uptake would have allowed us to better judge the effects of RPM in these patient populations. Third, since patients could be prescribed RPM at any time during the study, the duration of use varied and was brief in some cases. Longer follow-up may have shown different findings. Fourth, we did not survey patients, so cannot say what factors were barriers or facilitators to initial RPM prescription and its use once prescribed. Fifth, improvement in BP outcomes through RPM acts partially through better adherence to antihypertensive medications by patients; unfortunately, we did not collect information about antihypertensive medication adherence here. At last, the effects observed in patients on Medicare insurance may not be generalizable to the general population of patients with hypertension.

## CONCLUSION

At the two pilot practices, RPM uptake was modest even in uncontrolled hypertensive patients and highly variable across pilot physicians. Despite low uptake, compared with matched cohorts, we observed higher prevalence of achieving the Controlling High BP quality measure at 6 months in patients at practices where RPM was available. However, the mechanisms through which this occurred are not entirely clear. More work is needed to investigate the facilitators and barriers to optimal RPM use in primary care.

## Supplementary Material

ooac111_Supplementary_Data

## Data Availability

The data underlying this article cannot be shared publicly to respect the privacy of the individuals that participated in the study. A deidentified dataset will be shared on reasonable request to the corresponding author.
