# Supplementary material for: Remote physiologic monitoring for hypertension in primary care: a prospective pragmatic pilot study in electronic health records using propensity score matching
Source: JAMIA Open. 2023 Jan 31;6(1):ooac111. doi: 10.1093/jamiaopen/ooac111 (PMC9890085; doi:10.1093/jamiaopen/ooac111)

**Supplemental Material for Manuscript Titled:** Remote Physiologic Monitoring for Hypertension in Primary Care: A Prospective Pragmatic Pilot Study in Electronic Health Records Using Propensity Score Matching

**Short Title:** Remote Blood Pressure Monitoring in Primary Care

**Authors:** Lucia C. Petito, PhD^1^, Lauren Anthony, MPH^2^, Yaw Peprah, MPH^3^, Ji Young Lee, MS^3^, Jim Li, PhD^4^, Hironori Sato, PhD^5^, Stephen D. Persell, MD, MPH^3,6^

**Affiliations:**

1 Division of Biostatistics, Department of Preventive Medicine, Feinberg School of Medicine, Northwestern University, Chicago, IL, USA

2 Northwestern Medical Group Quality and Patient Safety, Northwestern Memorial Healthcare, Chicago, IL, USA

3 Division of General Internal Medicine and Geriatrics, Department of Medicine, Feinberg School of Medicine, Northwestern University, Chicago, IL, USA

4 Global Medical Affairs, Omron Healthcare, Co., Ltd., Kyoto, Japan

5 Product Innovation Department, Technology Development HQ, Omron Healthcare, Co., Ltd., Kyoto, Japan

6 Center for Primary Care Innovation, Institute for Public Health and Medicine, Feinberg School of Medicine, Northwestern University, Chicago, IL, USA

**Corresponding Author**: Lucia Petito, 680 N Lake Shore Drive Suite 1400, Chicago, IL 60611, [lucia.petito@northwestern.edu](mailto:lucia.petito@northwestern.edu)

**Key Words:** Remote physiologic monitoring, blood pressure, hypertension management

**Conflict of Interest:** Northwestern investigators (LCP, LA, YP, JYL, SDP) reported research funding from Omron Healthcare Co. Ltd. Dr. Li reported salary and reimbursement for travel from Omron Healthcare Co Ltd. Dr. Sato reported salary and reimbursement for travel from Omron Healthcare Co. Ltd. No other disclosures were reported.

**List of Material for Supplement**

Table S1. Identification of study variables from Northwestern’s Enterprise Data Warehouse

Table S2. Matching characteristics before and after imputation.

Table S3. Comparison of In-Office Versus Remote BP Measurements among Remote Monitoring Patients Who Transmitted at Least 12 Remote Readings During the Study Period Using Most Recent Office Visit and Most Recent Remote BP Measurement using A) AHA 2017 criteria and B) older criteria: November 2020 – May 2021.

Table S4. Results from Clinician User Experience Survey

Figure S1. Screenshots of order set implemented in Epic

Figure S2. Visualization of propensity score distribution for varying matching ratios in uncontrolled hypertensive population.

Figure S3. Visualization of propensity score distribution for varying matching ratios in general hypertensive population.

Table S1. Identification of study variables from Northwestern’s Enterprise Data Warehouse

| **Variable** | **Data source from EPIC** |
| --- | --- |
| Sex | Patient demographics |
| Ethnicity | Patient demographics |
| Race | Patient demographics |
| Age | Patient demographics |
| Language | Patient demographics |
| Counts of patient portal utilization | Encounters |
| Count of in-person/telehealth visits | Encounters |
| Counts of telephone encounters | Encounters |
| Diagnosed hypertension | Problem list and Encounter diagnosis |
| Office vitals (BP, HR, WT) | Flowsheets |
| Remotely measured vitals (BP, HR, WT) | Flowsheets |
| Orders of RPM device | Ordered procedure |

Table S2. Matching characteristics before and after imputation

|  |  | Average SBP 0-5 months prior to study start | | | Average DBP 0-5 months prior to study start | | | Average SBP 6-12 months prior to study start | | | Average DBP 6-12 months prior to study start | | |
| --- | --- | --- | --- | --- | --- | --- | --- | --- | --- | --- | --- | --- | --- |
|  |  | % missing | Observed | Imputed | % missing | Observed | Imputed | % missing | Observed | Imputed | % missing | Observed | Imputed |
| Uncontrolled Hypertensive Population | Pilot Practices | 54 (26.1) | 151.5 (10.4) | 151.7 (10.1) | 54 (26.1) | 78.9  (8.4) | 79.7 (8.1) | 64 (30.9) | 149.2 (13.8) | 148.5 (12.2) | 64 (30.9) | 79.7  (8.4) | 79.7  (8.4) |
|  | Matched Controls | 196 (23.7) | 151.5 (11.0) | 151.6 (10.8) | 196 (23.7) | 79.8  (9.2) | 79.7 (9.1) | 277 (33.5) | 148.6 (13.3) | 148.4 (11.5) | 277 (33.5) | 79.9  (9.1) | 79.4  (8.6) |
| General Hypertensive Population | Pilot Practices | 650 (27.6) | 130.8 (14.8) | 131.3 (14.7) | 650 (27.6) | 74.7  (8.4) | 74.9 (8.3) | 910 (38.6) | 131.4 (15.3) | 131.3 (14.1) | 910 (38.6) | 74.8  (8.5) | 74.8  (8.0) |
|  | Matched Controls | 1259 (26.7) | 131.4 (15.0) | 131.4 (15.0) | 1259 (26.7) | 74.9  (8.7) | 74.9 (8.8) | 1903 (40.4) | 131.9 (15.4) | 131.5 (13.9) | 1903 (40.4) | 75.0  (9.0) | 75.1  (8.3) |

Abbreviations: SBP: systolic blood pressure; DBP: diastolic blood pressure

**Table S3.** Comparison of In-Office^a^ Versus Remote^b^ BP Measurements among Remote Monitoring Patients Who Transmitted at Least 12 Remote Readings During the Study Period Based on Most Recent Office Visit and Most Recent Remote BP Measurement using A) AHA 2017 criteria and B) older criteria: November 2020 – May 2021.

|  |  | **RPM Measurement^b^** | |  |  |
| --- | --- | --- | --- | --- | --- |
|  |  | ≥130/80 | <130/80 | Total |  |
| **In-Office Measurement^a^** | ≥130/80 | 40 (49.4) | 28 (34.6) | 68 |  |
|  | <130/80 | 6 (7.4) | 7 (8.6) | 13 |  |
|  | Total | 46 | 35 | 81 |  |

|  |  | **RPM Measurement^b^** | |  |
| --- | --- | --- | --- | --- |
|  |  | ≥135/85 | <135/85 | Total |
| **In-Office Measurement^a^** | ≥140/90 | 11 (13.6) | 27 (33.3) | 38 |
|  | <140/90 | 12 (14.8) | 31 (38.3) | 43 |
|  | Total | 23 | 58 | 81 |

a When multiple blood pressure measurements were available on the same day, their minimum was used. In-office visits within 1 year of study end were included.

b Value used was most recent transmitted remote BP measurement.

Table S4. Responses to clinician user experience survey.

| **Questions** | **Possible Responses** | **N = 4 Physicians** |
| --- | --- | --- |
| *Have you ordered remote patient monitoring for hypertension since the pilot began in November?* | | |
|  | Yes | 3 (75%) |
|  | No | 1 (25%) |
| *If yes, about how many patients have you ordered remote patient monitoring for since the pilot began?* | | |
|  | Mean | 5 |
| *Have you ordered RPM for a patient and received blood pressure results in Epic?* | | |
|  | Yes | 3 (75%) |
| *If yes, about how many different patients have you received RPM blood pressure results for in Epic?* | | |
|  | Mean | 5 |
| *If yes, in response to RPM blood pressure values in Epic, have you…* | | |
| *…changed any patient’s medical treatment?* | | |
|  | Yes | 2 (67%) |
|  | Don’t know | 1 (33%) |
| *…had a staff member call the patient?* | | |
|  | Yes | 2 (67%) |
|  | No | 1 (33%) |
| *…communicated directly with a patient in Mychart?* | | |
|  | Yes | 3 (100%) |
| *…telephoned a patient directly?* | | |
|  | Yes | 2 (67%) |
|  | No | 1 (33%) |
| *…had a patient schedule an earlier appointment?* | | |
|  | Yes | 3 (100%) |
| *If yes, have you or your team logged any minutes spent managing patients with RPM for billing purposes?* | | |
|  | My team member(s) have logged minutes | 1 (33%) |
|  | Don’t know | 2 (67%) |
| *If yes, for blood pressure results returned from RPM, who is the primary person who reviews the blood pressure values?* | | |
|  | I am | 1 (33%) |
|  | Nurse | 2 (67%) |
| *Thinking about hypertensive Medicare patients you are seeing now…* | | |
| *… for patients for whom you are uncertain about whether their blood pressure is controlled, how likely are you to order RPM?* | | |
|  | Very unlikely | 1 (25%) |
|  | Somewhat likely | 1 (25%) |
|  | Very likely | 2 (50%) |
| *…for patients for whom you believe their blood pressure is uncontrolled, how likely are you to order RPM?* | | |
|  | Very unlikely | 1 (25%) |
|  | Unlikely | 1 (25%) |
|  | Very likely | 2 (50%) |
| *…for patients for whom you believe their blood pressure is controlled in the office but unknown at home, how likely are you to order RPM to look for masked hypertension?* | | |
|  | Very unlikely | 1 (25%) |
|  | Unlikely | 2 (50%) |
|  | Very likely | 1 (25%) |
| *How satisfied are you with the RPM ordering and set up process?* | | |
|  | Very satisfied | 1 (25%) |
|  | Satisfied | 2 (50%) |
|  | Don’t know | 1 (25%) |
| *How easy or difficult is it to set up a new patient with RPM?* | | |
|  | Very easy | 1 (25%) |
|  | Somewhat easy | 2 (50%) |
|  | Don’t know | 1 (25%) |
| *For hypertensive Medicare patients you have seen recently but did not order RPM, reasons for not ordering were…* | | |
|  | Concern about cost to patient | 4 (100%) |
|  | Concern about extra work for me to receive and respond to home blood pressures | 1 (25%) |
|  | Concern about extra work for team to receive and respond to home blood pressures | 2 (50%) |
|  | Patient declined | 1 (25%) |
|  | Patient already home monitoring | 4 (100%) |
|  | Blood pressure well-controlled | 3 (75%) |
| *How satisfied are you with the way you receive results back from patients who use the RPM process?* | | |
|  | Very satisfied | 1 (25%) |
|  | Satisfied | 2 (50%) |
|  | Don’t know | 1 (25%) |
| *Considering the RPM results you have received, indicate your level of agreement with each statement.* | | |
| *RPM results return too often.* | | |
|  | Strongly agree | 1 (25%) |
|  | Somewhat agree | 2 (50%) |
|  | Neutral | 1 (25%) |
| *Results do not return often enough.* | | |
|  | Neutral | 1 (25%) |
|  | Somewhat disagree | 2 (50%) |
|  | Strongly disagree | 1 (25%) |
| *It is helpful to receive very high or very low values right away.* | | |
|  | Somewhat agree | 2 (50%) |
|  | Neutral | 2 (50%) |
| *It is easy to use the information that returns in my Epic inbox.* | | |
|  | Strongly agree | 2 (50%) |
|  | Neutral | 2 (50%) |
| *It is too time consuming for me or my office to respond to RPM results.* | | |
|  | Somewhat agree | 1 (25%) |
|  | Neutral | 2 (50%) |
|  | Somewhat disagree | 1 (25%) |
| *When you view an individual patient's blood pressure data, how easy is it to tell if the patient's hypertension is controlled?* | | |
|  | Very easy | 1 (25%) |
|  | Somewhat easy | 2 (50%) |
|  | Don’t know | 1 (25%) |
| *Considering your experience with RPM for hypertension, if 1 is don't recommend at all and 10 is strongly recommend, please indicate how strongly you recommend RPM for other NM primary care practices?* | | |
|  | Median (Range) | 7 (1-10) |
| *Considering your experience with RPM for hypertension, what do you recommend NM do for other primary care practices?* | | |
|  | Roll out RPM with minor changes/improvements | 3 (75%) |
|  | Don’t know | 1 (25%) |
| What changes could NM make to improve the experience of using RPM for clinicians or patients?  “Not sure one high reading should cause a panic alert, don't know the context of when the patient took reading and if it is a resting reading or not”.  “Dedicated triage RN to screen incoming readings, collect data, and notify MD when needed at which time patient should schedule in person follow up appointment might be an ideal workflow as opposed to the MD getting all this primary raw data in the inbox first without triage”.  “Recommend having a dedicated RN or LPN in the office or within the region to review the BP data and forward the summation of BP readings which is outside the BP parameters for physician's input. Therefore, limiting the BP readings reviewed by physicians to those that need attention or action”. | | |
|  |  |  |
| What questions should we make sure new physicians know about RPM?  “How to set the BP reading result frequency (Q 2wks or Q 4wks) vs readings since some patients check their BP readings 2-4 times a day (despite recommending BP check Q daily or BID)”.  “Ease of entering order; ability to generate billing and specifics for how to do this”.  “Path for monitoring within each office and who might see extra work | | |
|  |  |  |


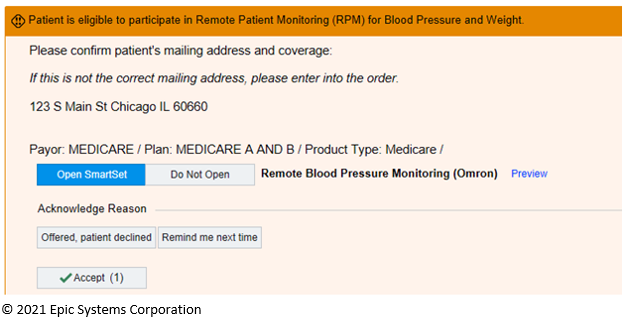
Figure S1. Screenshots of order set implemented in Epic


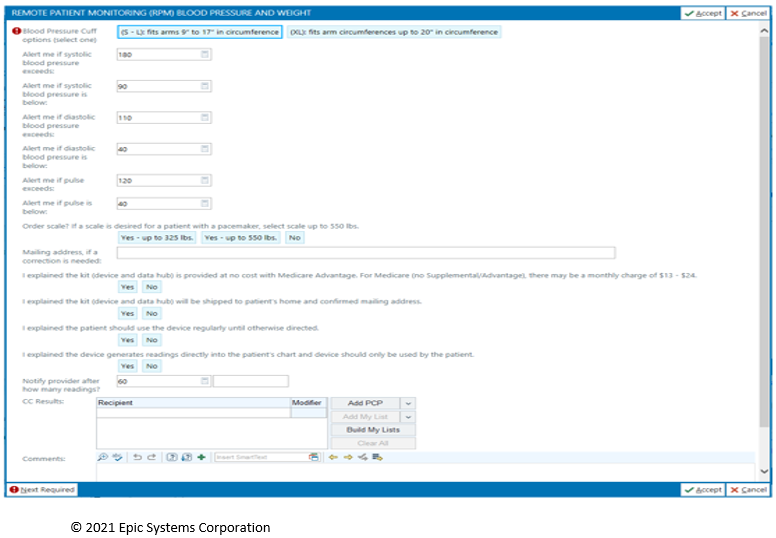


**Figure S2.** Visualization of propensity score distribution for varying matching ratios in uncontrolled hypertensive population.


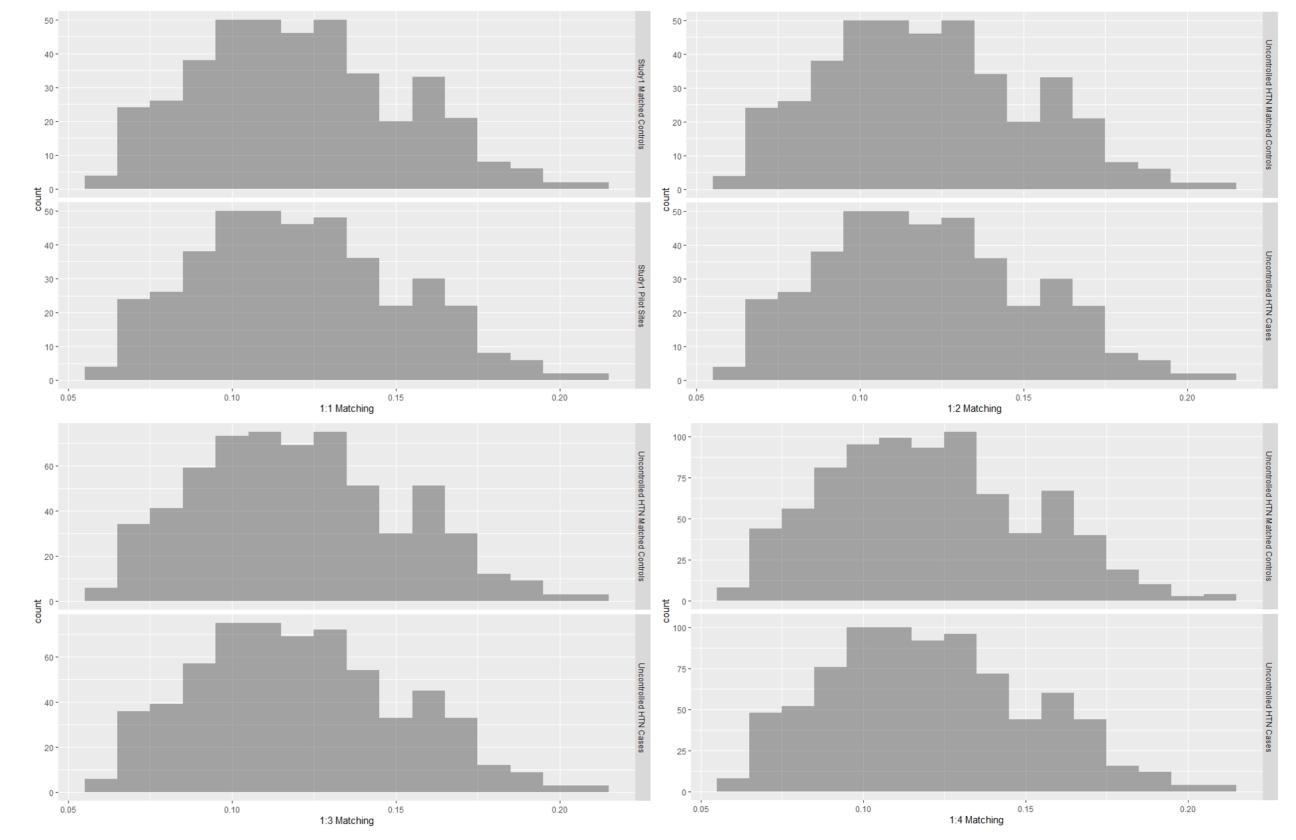


**Figure S3.** Visualization of propensity score distribution for varying matching ratios in general hypertensive population.


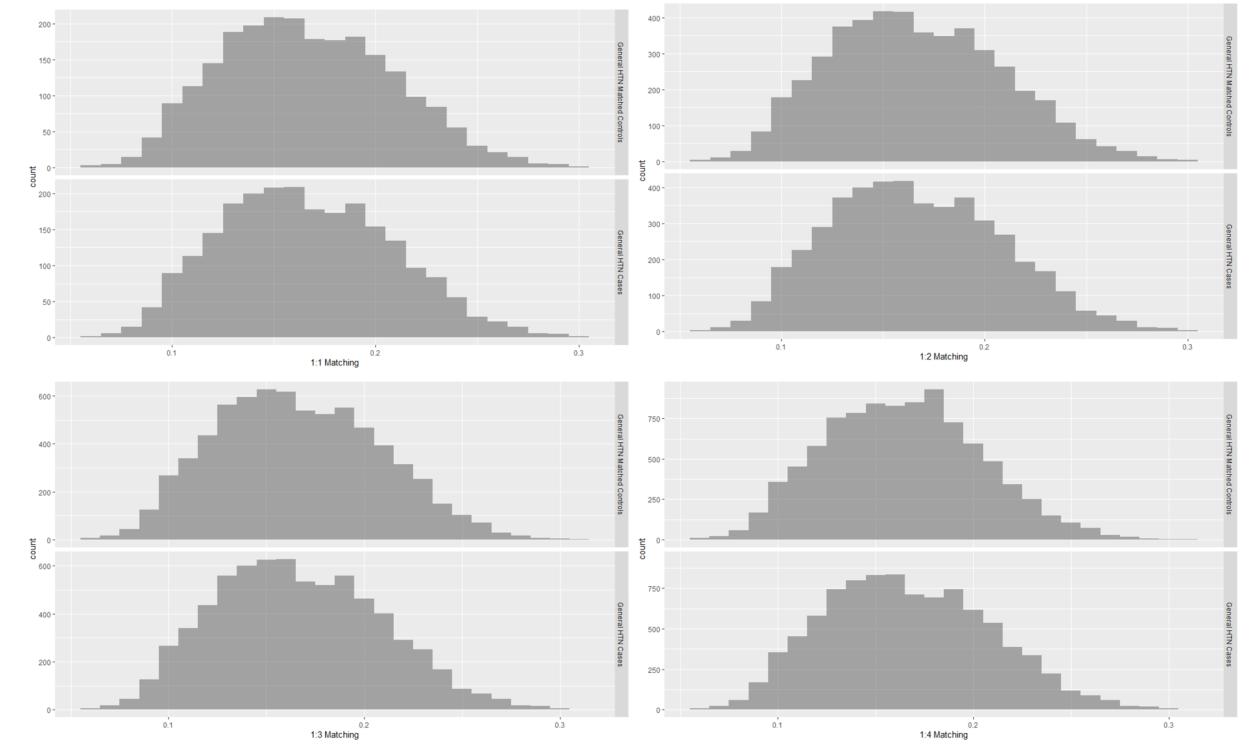

Supplement: ooac111_Supplementary_Data [file ooac111_supplementary_data.docx]
